# Role of Nitric Oxide and CCAAT/Enhancer-Binding Protein Transcription Factor in Statin-Dependent Induction of Heme Oxygenase-1 in Mouse Macrophages

**DOI:** 10.1371/journal.pone.0064092

**Published:** 2013-05-22

**Authors:** Charbel A. Mouawad, May F. Mrad, Moustafa Al-Hariri, Hiba Soussi, Eva Hamade, Jawed Alam, Aïda Habib

**Affiliations:** 1 Department of Biochemistry and Molecular Genetics, American University of Beirut, Beirut, Lebanon; 2 Génomique et Santé, Lebanese University, Hadath, Lebanon; 3 Department of Molecular Genetics, Ochsner Clinic Foundation, New Orleans, Louisiana, United States of America; 4 Ochsner Clinical School - The University of Queensland School of Medicine, Brisbane, Queensland, Australia; Medical Faculty, Otto-von-Guericke University Magdeburg, Germany

## Abstract

The effect of statins on heme oxygenase-1 (HO-1) was compared in 2 murine cell lines, RAW 264.7 and J774A.1 cell lines, and in primary peritoneal macrophages of BALB/c or C57BL/6 mice. The role of endogenous nitric oxide and the type of transcription factors involved were explored. Simvastatin and fluvastatin induced HO-1. Pretreatment of cells with *l*-NMMA or 1400 W, two different nitric oxide synthase inhibitors, partially blocked statin-dependent induction of HO-1 in RAW 264.7 and J774A.1 but not in primary peritoneal macrophages. Induction of HO-1 by statins was dependent on p-38 MAP kinase activation in all types of macrophages. In RAW 264.7 cells, both statins increased the activity of reporter genes linked to the proximal 1.3 kbp promoter of HO-1 (EC_50_ of 1.4±0.3 µM for simvastatin and 0.6±0.03 µM for fluvastatin). This effect was significantly blocked by 1400 W (80±5.2% inhibition, p<0.02) and mevalonate, the direct metabolite of HMGCoA reductase. Gel retardation experiments implicated C/EBPβ, AP-1 but not USF, for both RAW 264.7 and primary peritoneal macrophages of C57BL/6 mice. Collectively we showed a differential role of endogenous nitric oxide between macrophage cell lines and primary macrophages and an effect of statins in the protection against inflammation by increasing HO-1 expression.

## Introduction

Statins are lipid-lowering agents that act as competitive inhibitors of the 3-hydroxy-3-methylglutaryl coenzyme A (HMG-CoA) reductase [1]. Studies have shown beneficial and protective effects of statins in reducing mortality and morbidity beyond their capacity to lower cholesterol. These pleiotropic effects vary from improvement of endothelial function to anti-thrombotic and anti-inflammatory properties [2]. In macrophages, it has been shown that statins decrease the production of proinflammatory cytokines such as interleukin (IL)-6, tumor necrosis factor (TNF)α [3], [4] and monocyte chemoattractant protein MCP-1 [5], expression of adhesion molecule, tissue factor [6] as well as cyclooxygenase-2 and prostaglandin synthesis [7]. Some of these beneficial effects, such as the improvement of endothelial function and anti-oxidant capacities were demonstrated *in vivo* [8]. Heme oxygenase-1 (HO-1) is the inducible isoform of heme oxygenase, a microsomal enzyme responsible for the oxidative degradation of heme. The resulting end products contribute to the anti-oxidant, anti-inflammatory and anti-apoptotic actions of HO [9], [10]. Many inducers have been reported for HO-1 including, pro and anti-inflammatory cytokines [11], lipopolysaccharide (LPS) [12] and nitric oxide (NO) [13]. HO-1 has been shown to be a downstream effector of IL-10 [14] and to play a role in the resolution of inflammation [15]. Stimulation of HO-1 expression by a large number of stimuli is regulated mainly at the transcriptional level which is controlled by many regulatory elements located in the proximal and distal sites of the HO-1 promoter gene 5′flanking region [16], [17]. Controversial roles of endogenous NO in HO-1 expression in RAW 264.7 cells were described [18], [19], [20]. However the intracellular pathways involved in this regulation in primary cells were not investigated. We have recently reported a role of C/EBPβ and δ and USF-1 and −2 in statin-dependent induction of HO-1 in NIH 3T3 fibroblasts [21]. Since macrophages are important players in inflammation and since statins were shown to have anti-inflammatory effects, we aimed to study the regulation of HO-1 and the mechanisms underlying this regulation in different types of macrophages, primary cells and murine cell lines, mainly by investigating the role of endogenous NO and the transcription factors involved.

## Experimental Procedures

### Materials

Cell culture media were from Lonza (Verviers, Belgium). Fetal bovine serum (FBS) and lipofectamine 2000® were obtained from Gibco (Invitrogen, New York, USA). Penicillin and streptomycin were purchased from Cambrex (Rockland, USA). Chemicals for electrophoresis and Bradford reagent were from Bio-Rad (Hercules, USA). Simvastatin, fluvastatin, FTI-277, GGTI 286, *l*-NMMA, 1400 W and SB203580 were from Calbiochem (San Diego, USA). Monoclonal anti-β actin, lipopolysaccharide 0111:B4, bovine serum albumin (BSA), N-(1-Naphtyl)- ethylendiamine dihydrochloride 98%, sulphanilamide and sodium nitrite were from Sigma Aldrich (St. Louis, USA). Farnesyl pyrophosphate (FPP) and geranylgeranyl pyrophosphate (GGPP) were from MP Biomedicals (Costa Mesa, CA, USA). Spermine NONOate (SPNO) was from Cayman chemicals (Ann Arbor, USA). Enhanced chemiluminescense (ECL) was from Amersham (General Electric Healthcare Life Sciences, USA) and Roche (Mannhein, Germany). Fugene 6® was from Roche. Modified Thioglycollate Medium Brewer was obtained from Becton Dickinson (Sparks, USA). Donkey anti-mouse or anti- rabbit antibodies conjugated to peroxidase were from Jackson Immunoresearch Laboratories (Pennsylvania, USA). Rabbit polyclonal antibodies against C/EBPα (14AA), C/EBPβ (C-19) and C/EBPδ (C-22) were purchased from Santa Cruz Biotechnology (Santa Cruz, CA, USA). C3 exoenzyme and GST-cyctonecrotic factor −1 were kind gifts of Dr. Jacques Bertoglio (INSERM U 749, Institut Gustave Roussy, Villejuif, France). DNA oligonucleotides were obtained from TIB Molbiol (Berlin, Germany). ELISA assays for murine IL-6 and TNFα were from eBioscience (San Diego, CA). Sn-protoporphyrin was from Frontier Scientific Inc. (Logan, UT, USA). HO-1 promoter regions (1.3 kbp and 15 kbp-luciferase constructs) were developed as previously described [17], [22]. All other chemical reagents were purchased from Amresco (Solon, USA).

### Cells

RAW 264.7 and J774A.1 cells were obtained from ATCC (Manassas, VA, USA). Elicited mouse peritoneal macrophages (eMPM) were harvested from BALB/c or C57BL/6 mice. Approval for use of animals was obtained from the Institutional Animal Care and Use Committee of the American University of Beirut (permit # 06-10-026). Mice were injected i.p. with 1 ml of sterile 3.85% thioglycollate broth for 48 to 72 hours and the peritoneal cavity was washed with 10 ml of RPMI 1640 culture medium containing 2% FBS. Macrophages were allowed to adhere for 2 hours and washed twice before treatment.

### Cell Culture

Murine RAW 264.7 and J774A.1 cells were grown in DMEM culture medium supplemented with 20 mM HEPES buffer (pH 7), 10% FBS, 2 mM L-glutamine, 100 U/ml penicillin and 100 µg/ml streptomycin, at 37°C in 10% CO_2_. For treatment, cells were cultured in 12-well plates for western blot analysis (1×10^6^ cell/well) or transfection experiments (0.5×10^6^ cell/well), and in 60 mm dishes for electrophoretic mobility shift assay. Subconfluent cells were treated in serum-free culture medium alone or in the presence of simvastatin or fluvastatin for 24 hours and various concentrations of LPS. In some experiments, NO synthase inhibitors, *l-*NMMA (1 mM) or 1400 W (10 µM), or p38 MAP kinase inhibitor, SB203580, (10 µM) were added 30 minutes prior to the addition of statins. The concentration of DMSO did not exceed 0.2%. The cellular toxicity by statins was evaluated by neutral red assay [23] and did not exceed 10%. At the end of treatment, supernatants of cell culture were kept at −70°C until IL-6, TNFα and nitrite were measured.

### Western Blot Analysis

At the end of the treatment, cells were washed with phosphate-buffer saline and lysed in 300 µl of lysis buffer (20 mM Tris/HCl, pH 8, containing 1 mM EDTA, 1% Triton and protease inhibitor cocktail). Protein content was determined using the Bradford Protein Assay with BSA as standard. Immunoblotting was performed as described previously [24] using 20 µg of total protein. Blotting was performed using specific polyclonal antibodies against HO-1 (1/2000), HO-2 (1/1000) [24], inducible NOS (1/1000) [25] and monoclonal antibody against β-actin (1/2000) (Sigma-Aldrich) and immunoblotting was carried out as previously described [21].

### Determination of Nitrite Concentration

Nitrite concentrations were determined according to the Griess method with sodium nitrite used as a standard [26]. The optical density was immediately measured at 540 nm on a 96-well plate reader (Multiskan EX, Thermo Electron). Nitrite concentrations were obtained in µM.

### Measurement of IL-6 and TNFα Concentrations

Mouse IL-6 and TNFα were measured in the supernatants of cell culture according to the manufacturer’s instruction (eBioscience, San Diego, CA). Results were expressed in pg/ml.

### Electrophoretic Mobility Shift Assay

The sequences of the primers used to prepare the double strand DNA probe were reported earlier and the experiments were performed as previously described [21]. Briefly, 5 µg of nuclear extract and 5×10^4^ cpm of the desired probe were allowed to bind in 20 mM Tris/HCl, pH 8, containing 120 mM KCl, 25 mM MgCl_2_, 2 mM EDTA, 25% Glycerol and 2 mM DTT for 30 min on ice. For supershift experiments, antibodies were incubated with the nuclear extracts for 30 minutes on ice before the addition of the labelled probe. Binding activities were measured by Storm 860® phosphorImager.

### Reporter Assay

RAW 264.7 cells were seeded at 0.5×10^5^ cells/well in 12-well plates one day before transfection. pHO-1-1.3luc or pHO-1-15luc gene promoter vectors were transiently transfected by FuGene 6® (Roche) according to the manufacturer’s instructions. Cells were treated 24 hours post-transfection for an additional 24 hours with different concentrations of statins. In some experiments, cells were incubated for 30 minutes with 10 µM 1400 W prior to the addition of statins. Luciferase activity was evaluated as described in [21]. Results were normalized to the protein concentration. Fold activation was calculated by dividing the corrected values of treated cells with the control value, which was attributed a value of 1.

### Data Analysis

Autoradiograms obtained from western blot analyses were scanned using Epson 1680 pro scanner and densitometric analysis was performed using Scion NIH software (Scion Corp., Frederick MD). Results are shown as average ± S.E.M. Data was analyzed by student’s *t*-test (Sigma Stat®, Systat Software, Inc., San Jose, CA, USA).

## Results

### Effect of Statins on HO-1 Expression in Murine Macrophages

**Figure 1:**
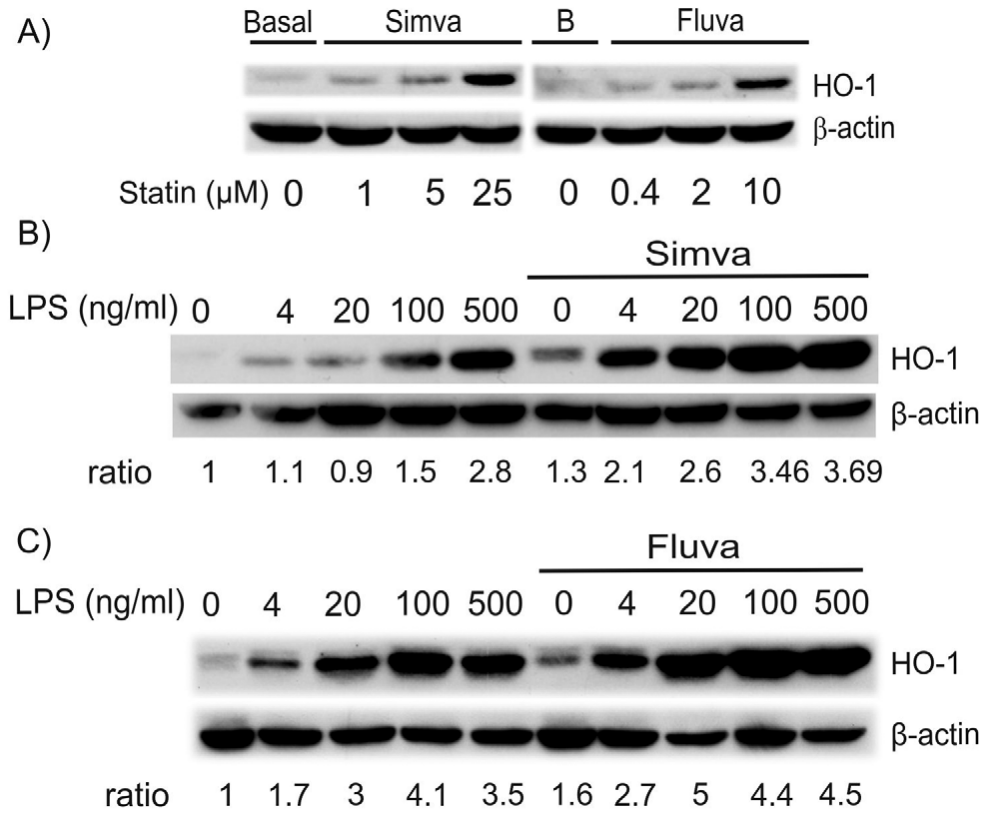
HO-1 induction by statins in RAW 264.7 cells. Cells were incubated for 24 hours with simvastatin or fluvastatin. 20 µg of total proteins were loaded on a 12% SDS polyacrylamide gel and blotted for HO-1 and then stripped and blotted for β-actin. HO-1 band intensities were measured by densitometry and normalized to the intensity of β-actin bands. Fold increase was calculated as a ratio of the result of stimulated cells to that of the control cells. (A) Dose-response of simvastatin or fluvastatin in RAW 264.7 cells. The western blot is representative of 4 experiments with similar results. B corresponds to basal untreated cells. (B &C) Effect of statins on LPS induced HO-1 expression. RAW 264.7 cells were incubated for 24 hours with increasing concentrations of LPS alone or in the presence of 25 µM simvastatin (B) or 10 µM fluvastatin (C). Results are representative of 3 experiments.

**Figure 2:**
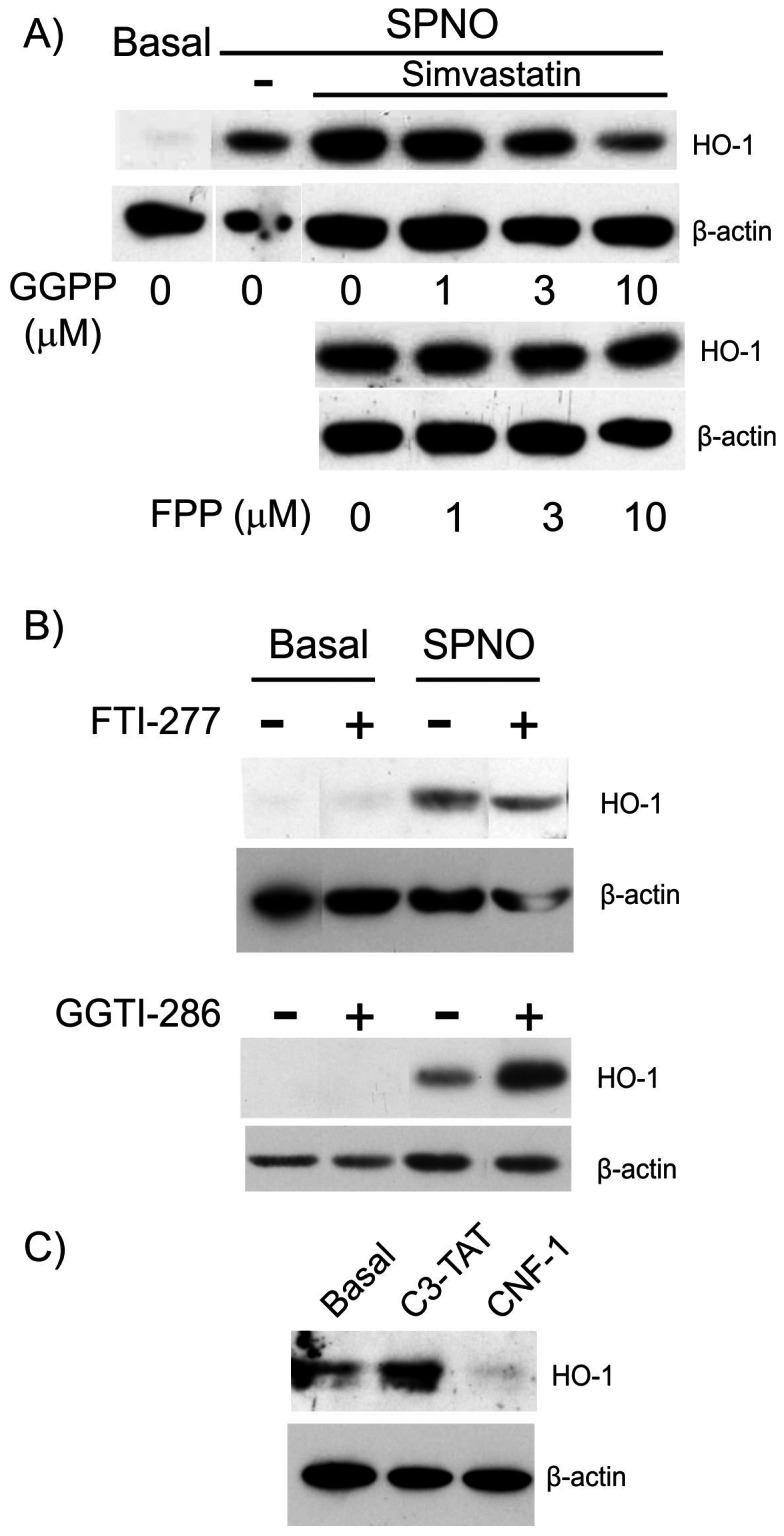
Role of prenylation in simvastatin-dependent induction of HO-1. (A) RAW 264.7 cells were co-treated with 25 μΜ simvastatin and increasing concentrations of GGPP and FPP for 24 hours in the presence of 125 µM SPNO. Western blotting of HO-1 was performed as described in the legend for figure 1. Results are representative of 3 experiments for GGPP and 2 for FPP. (B) RAW 264.7 cells were incubated with 10 µM of GGTI-286 or FTI-277 for 24 hours. Results are representative of 3 experiments. (C) RAW 264.7 cells were treated with 5 µg/ml of C3-exoenzyme and 1 µg/ml of CNF-1 for 24 hours. Results are representative of 3 experiments with similar results.

We first checked that statins induced HO-1 in RAW 264.7 cells. Treatment of murine macrophage cell line RAW 264.7 for 24 hours with different concentrations of simvastatin (1, 5 and 25 µM) or fluvastatin (0.4, 2 and 10 µM) increased HO-1 expression (Figure 1A). Induction of HO-1 was slightly detectable at 5 µM simvastatin and 2 µM fluvastatin; maximal statistically significant induction was observed with 25 µM simvastatin and 10 µM fluvastatin (2.9±0.5 and 2.3±0.3 fold of HO-1 expression of control, mean ± S.E.M., n = 4, p<0.005 and p<0.01, *t*-test, for simvastatin and fluvastatin, respectively). We next examined the effect of co-treatment of macrophages with statins and lipopolysaccharide (LPS). LPS increased HO-1 expression in a dose-dependent manner (Figure 1). Treatment of cells with simvastatin (Figure 1B) or fluvastatin (Figure 1C) further amplified this expression. Strong additive effects between statins and LPS were evident at low concentrations of LPS, i.e. 4 and 20 ng/ml. We next checked the intermediates of the mevalonate pathway. SpermineNONOate or SPNO, a strong NO donor, was used as a control for testing the effect of isoprenoid derivatives. Induction of HO-1 by simvastatin+SPNO was abrogated after treatment with 10 µM geranylgeranyl-pyrophosphate but not with farnesyl-pyrophosphate (Figure 2A). Moreover, geranylgeranyltransferase inhibitor alone, GGTI-286 but not farnesyltransferase inhibitor, FTI-277, increased SPNO-induced HO-1 expression in RAW 264.7 cells suggesting a role of geranylgeranylated proteins in modulating HO-1 expression (Figure 2B). Inactivation of Rho A/C by C3- exoenzyme induced expression of HO-1 (Figure 2C) whereas activation of Rho members by cytonecrotic factor (CNF)-1 showed a decrease in HO-1 expression in the presence of statins (Figure 2C).

**Figure 3:**
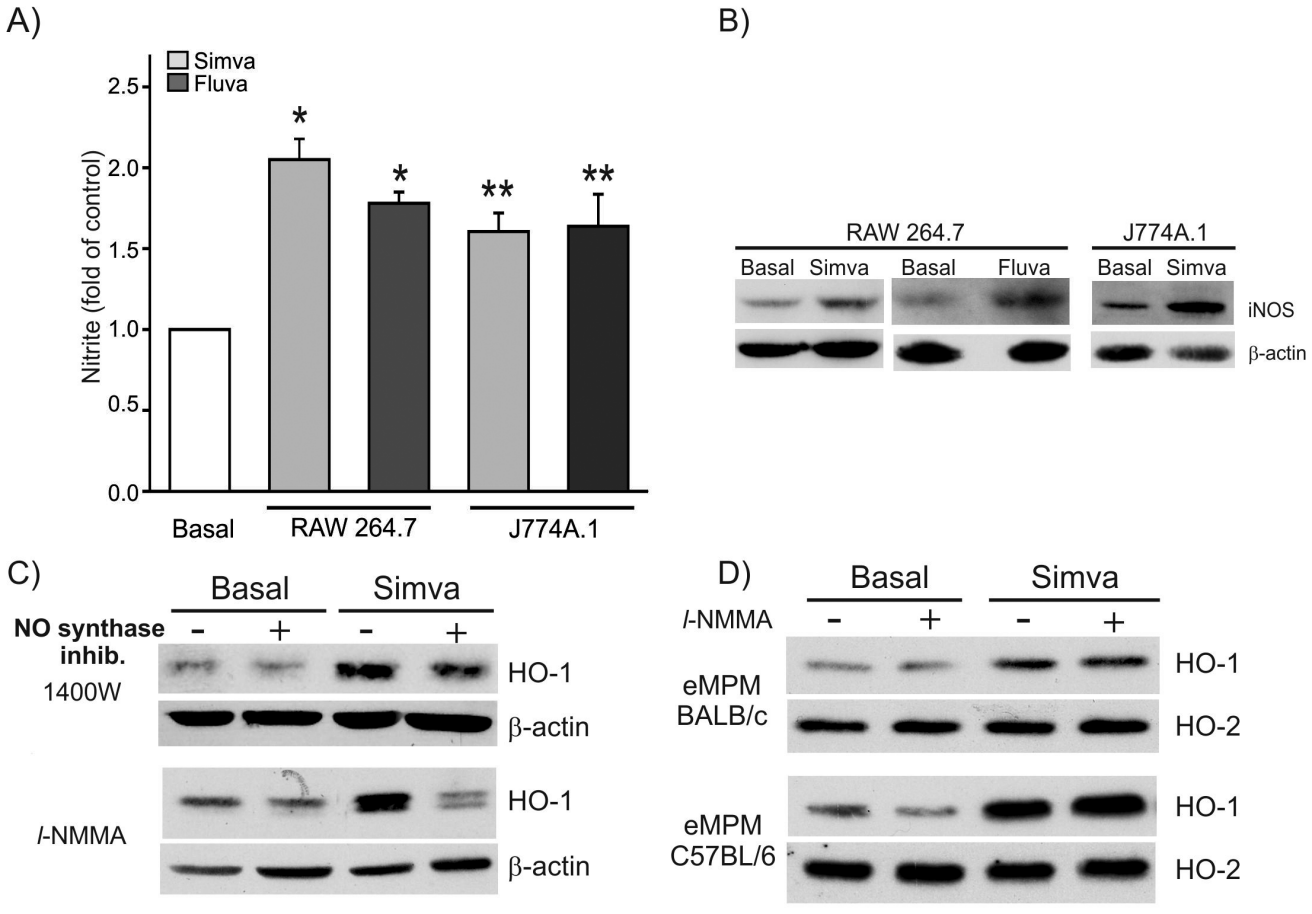
Role of NO and NO activity in statin-dependent induction of HO-1. (A) Nitrite formation in response to statins. RAW 264.7 or J774A.1 cells were incubated for 24 hours with 25 µM simvastatin or 10 µM fluvastatin. Nitrite was measured in the supernatant by the Griess method. Results correspond to mean ± S.E.M of the fold increase of nitrite release in treated RAW 264.7 (n = 17, *p<0.001, *t-test*) or J774A.1 cells (n = 10–12, **p<0.02, *t-test*) compared to untreated basal cells. (B) iNOS induction by statins. 40 µg of total proteins were loaded on a 7% SDS polyacrylamide gel and blotted for iNOS or β-actin. Results are representative of 2–3 experiments. (C) Effect of 2 NO synthase inhibitors on HO-1 expression in RAW 264.7 cells. Cells were pre-treated for 30 minutes with 10 µM 1400 W or 1 mM *l*-NMMA prior to the addition of 25 µM simvastatin for 24 hours. Results are representative of 3 experiments. (D) Effect of *l*-NMMA on HO-1 expression in eMPM cells. eMPM were prepared from BALB/c or C57BL/6 mice and treated as mentioned in the previous paragraph. Results are representative of 3 experiments for eMPM from BALB/c and 2 from C57BL/6.

We next investigated the role of endogenous NO formation in statin-dependent induction of HO-1 in different macrophages. We first analyzed the formation of nitrite, one of the breakdown products of NO, in response to statins. Simvastatin and fluvastatin were able to increase nitrite release in RAW 264.7 cells (10±0.6 µmol/L for basal untreated cells compared to 20.9±0.7 µmol/L for simvastatin and 19.1±0.5 µmol/L for fluvastatin, n = 23) and to induce iNOS protein expression with a fold increase over control of 1.5±0.2 for simvastatin and 1.4±0.1 for fluvastatin (n = 3–4, p<0.05, *t-*test) (Figure 3B). We studied in parallel the effect of statins on HO-1 expression in J774A.1 cells, another murine macrophage-like cell line. Similar findings to Raw 264.7 cells were obtained in statin and iNOS induction; statins increased nitrite release (5.2±0.6 µmol/L for basal untreated cells compared to 8.5±0.3 µmol/L for simvastatin and 8.2±0.2 for µmol/L for fluvastatin, n = 13) (Figure 3A) (1.5 fold induction of control, n = 2). To analyze the role of endogenous NO in statin-dependent induction of HO-1, we pretreated RAW 264.7 cells for 30 minutes with *l*-NMMA, a broad inhibitor of NO synthases or 1400 W, a more selective inhibitor of iNOS, prior to the addition of simvastatin. Figure 3C shows a significant reduction in HO-1 expression (35±4.7% for 1400 W and 64±3.2% for *l-*NMMA, (n = 3, p<0.01 and p<0.001, t-test, for 1400 W and *l*-NMMA, respectively) (Figure 3C). In these cells, *l*-NMMA and 1400 W blocked significantly nitrite formation in response to LPS (85±2% inhibition, n = 5 and 60.2±4.5% inhibition, n = 4, respectively). We performed similar experiments in primary murine elicited macrophages. No nitrite was detected by the Griess measurement method in eMPM from BALB/c mice in response to statins or LPS. Treatment of these cells with *l*-NMMA did not modify HO-1 induction in response to simvastatin (Figure 3D). Since macrophages from BALB/c are Th-2 responder cells with a major production of ornithine from *l*-arginine [27], we checked peritoneal macrophages from Th-1 responder mouse strain C57BL/6, which produce NO when activated by LPS. In these cells, treatment with LPS resulted in a significant increase of nitrite (2–10 µM, n = 3) compared to undetectable levels in untreated macrophages. Pretreatment with *l*-NMMA did not modify HO-1 induction in response to simvastatin although it blocked significantly nitrite formation (88±3.2% inhibition, n = 3, of LPS treated cells) (Figure 3D).

**Figure 4:**
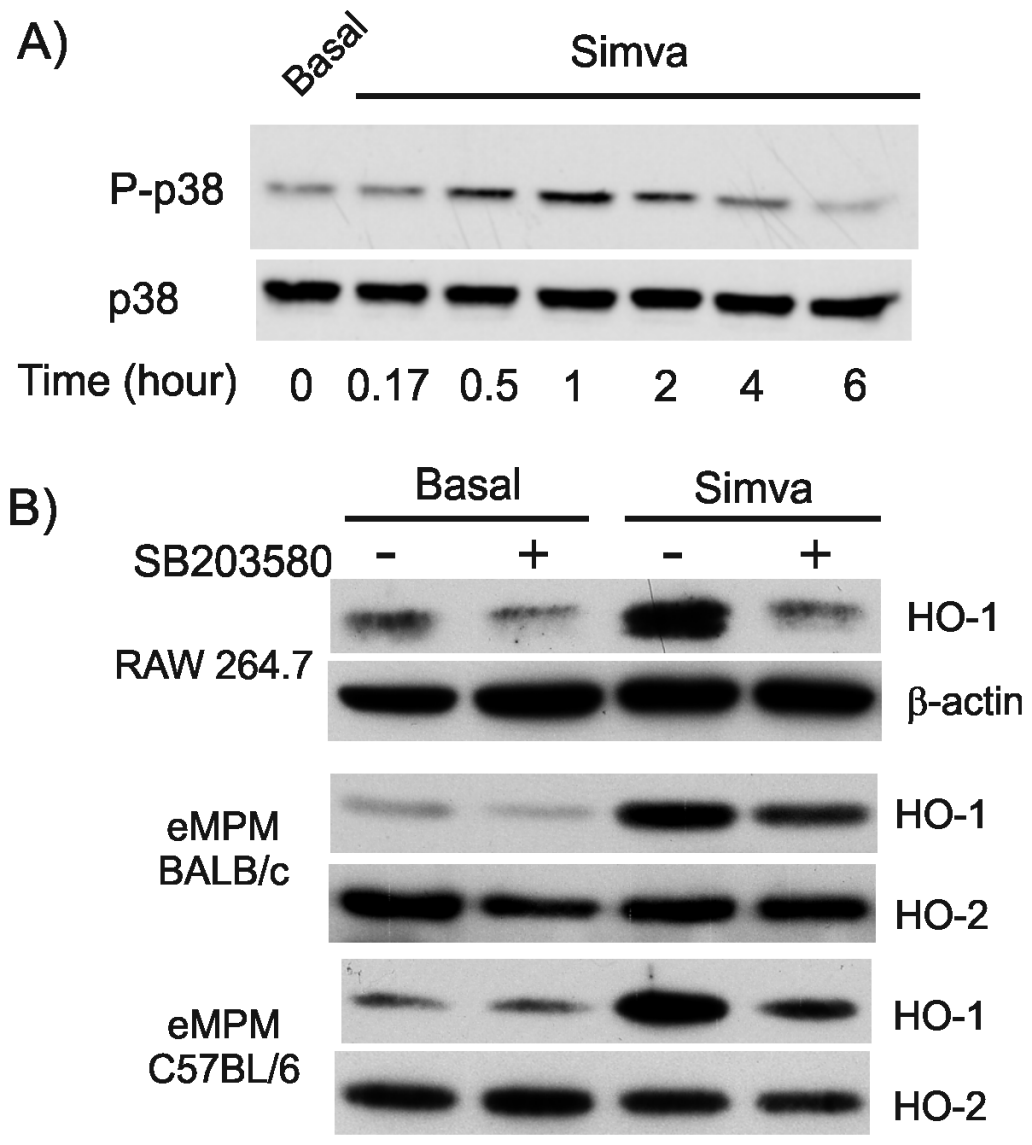
Implication of P38 MAP kinase pathway in statin-dependent induction of HO-1. (A) Phosphorylation of P38 by 25 µM simvastatin at different times in RAW 264.7 cells. Results are representative of 3 experiments. (B) Effect of p38 MAP kinase inhibitor, SB203580 on HO-1 induction in RAW 264.7 and eMPM. Cells were pretreated for 30 minutes with 10 µM of SB203580 prior to the addition of 25 µM of simvastatin for 24 hours. Western blotting of HO-1 was performed as indicated earlier. Results are representative of 3 experiments.

Since p38 MAP kinase was shown to play an important role in HO-1 induction in response to different inducers [28] and since statins were shown to modulate p38 MAP kinase, we compared the involvement of p38 MAP kinase activity among the different macrophages. Treatment of RAW 264.7 cells with simvastatin increased p38 MAP kinase with a peak after 1 hour treatment (Figure 4A) whereas 10 µM SB203580, a p38 MAP kinase inhibitor, decreased significantly simvastatin-dependent induction of HO-1 in all three macrophages models providing evidence for a role of p38 MAP kinase in HO-1 expression in all macrophages primary and cell lines (Figure 4B).

### Effect of Statins on Cytokines Release in eMPM

IL-6 and TNFα concentrations were measured in the supernatant of eMPM cells prepared from C57BL/6. Treatment of cells with 100 ng/ml LPS for 24 hours resulted in an increase in cytokines production in cells (11540±3210 pg/ml of IL-6 (n = 5) and 84350 pg/ml of TNFα (n = 2) compared to undetectable levels in controls). Pretreatment of cells with 25 µM simvastatin reduced IL-6 production by 40±6% (n = 5) and TNFα by 50% ±3% (n = 3). Sn-protoporphyrin (50 µM) inhibited LPS-dependent IL-6 by 81±3% (n = 4) but not TNFα synthesis and reversed the inhibition observed for TNFα+simvastatin but not IL-6+ simvastatin (96% ±5%, n = 3).

### Transcriptional Regulation of HO-1 Promoter Gene by Statins

**Figure 5:**
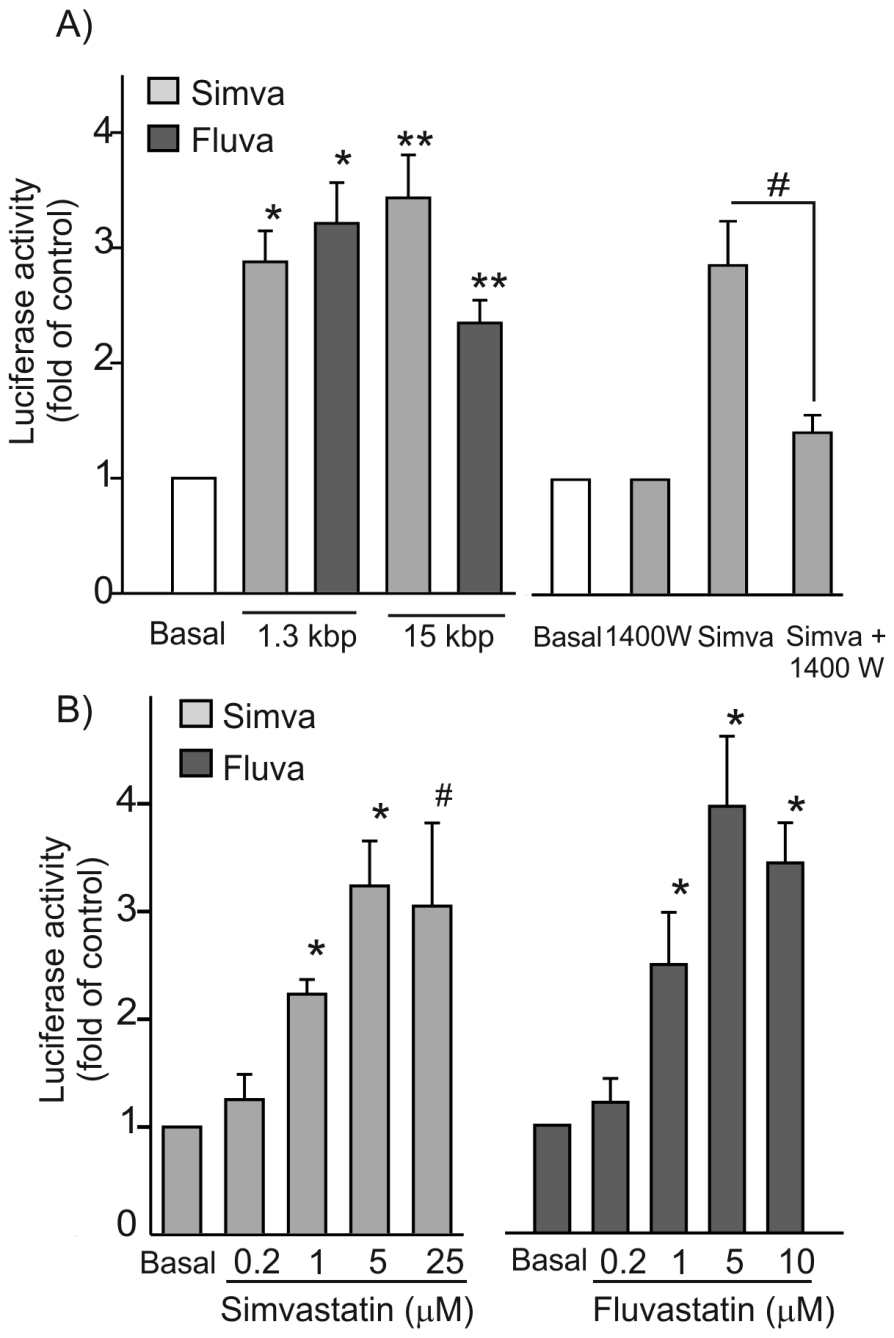
Regulation of HO-1 promoter activity by statins in RAW 264.7 cells. (A, left panel). Cells were transfected with 1.3 or 15 kbp HO-1 Luciferase gene promoter vectors and treated 24 hours later with 25 µM simvastatin or 10 µM fluvastatin for an additional 24 hours. Luciferase activity was measured and normalized to total protein and expressed as fold increase compared to untreated (control) cells attributed the value of 1. Results represent mean ± S.E.M. of 3–6 different experiments. (A, right panel) Effect of 1400 W on the 1.3 kbp HO-1 promoter activity by simvastatin in RAW 264.7 cells. Cells transfected with the 1.3 kbp HO-1 Luciferase promoter were incubated 24 hours post-transfection with 10 µM 1400 W for 30 minutes prior to the treatment with 25 µM simvastatin for 24 hours. Results represent 3 different experiments. (B) Dose-response of simvastatin and fluvastatin on 1.3 kbp HO-1 Luciferase promoter activity. Cells were transfected as described earlier and treated with different concentrations of statins for 24 hours. Results represent 3 different experiments.

**Figure 6:**
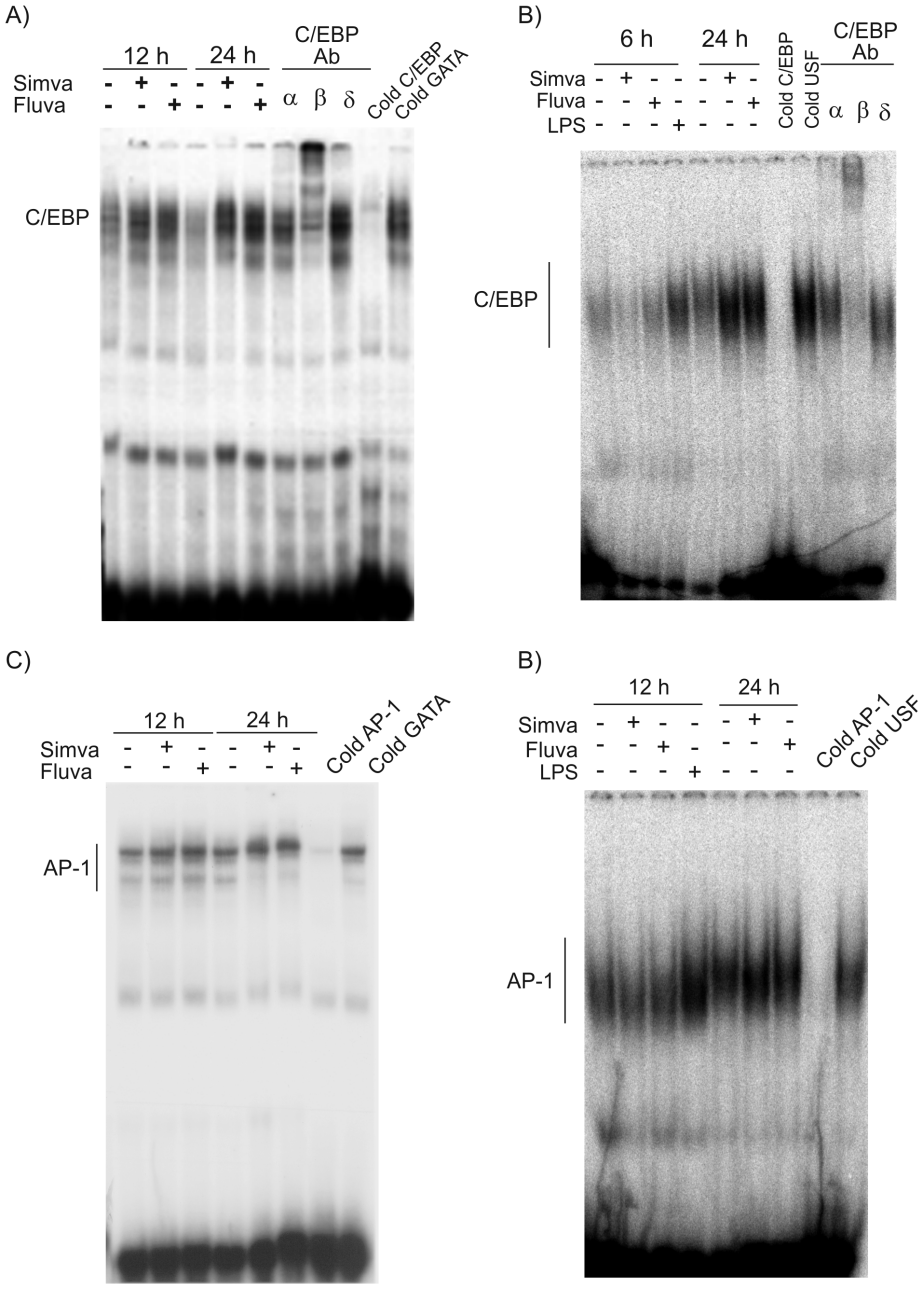
Transcriptional regulation of HO-1 by statins in mouse macrophages. Gel retardation analyses for C/EBP in (A) Raw 264.7 cells or (B) eMPM of C57BL/6 in response to statins. Cells were incubated with 25 µM simvastatin or 10 µM fluvastatin for 6, 12 or 24 hours. Nuclear extracts were prepared and incubated with labeled double stranded DNA C/EBP as described in Materials and Methods. DNA-protein complexes were detected after migration on non denaturing acrylamide. Storm PhosphorImager was used to detect radioactive bands. Competition was done with unlabeled C/EBP, GATA, or USF double-stranded DNA added in excess in 24 hour fluvastatin treated cells. For supershift experiments, nuclear extracts were incubated for 30 min in the absence or presence of 2 µg of C/EBPα, β or δ antibodies prior to the addition of the labeled probe. Results are representative of 3 different experiments with similar results showing a formation of C/EBP-β- protein complex. (C & D) Gel retardation analyses for AP-1-protein complex formation in response to statins at 12 and 24 hours in Raw 264.7cells and eMPM of C57BL/6, respectively Competition was done with unlabeled AP-1, GATA or USF double-stranded DNA added in excess of 24 hours- fluvastatin treated cells. Results are representative of 3 different experiments with similar results.

Previous studies have shown in RAW 264.7 an ability of statins to induce AP-1 and to modulate NFκB in macrophages [7], [20]. We have previously shown that statins increase HO-1 promoter activity in NIH 3T3 cells [21]. To investigate whether HO-1 induction by statins involved transcriptional regulation in this more physiologically relevant model, we initially studied the effect of statins on HO-1 promoter activity in RAW 264.7 cells. Cells were transfected with expression vectors encoding the murine HO-1 promoter genes of 1.3 kbp and 15 kbp [16]. As shown in Figure 5A left panel, 25 µM simvastatin increased the 1.3 kbp and the 15 kbp HO-1 promoter luciferase activity by 2.9±0.3 (n = 6, **P<0.001, paired *t*-test) and 3.2±0.4 (n = 3, *P<0.01, paired *t-*test), compared to untreated cells, respectively. 10 µM fluvastatin also increased reporter activity by 3.4±0.4 (n = 6, **P<0.001, paired *t*-test) and 2.4±0.2 (n = 3, *P<0.01, paired *t*-test) for 1.3 kbp and the 15 kbp HO-1 promoters, respectively. Pretreatment of cells with 200 µM mevalonate prior to statins addition resulted in an inhibition of statin-dependent 1.3 kbp HO-1 promoter activity by more than 68±6.6% (n = 3, p<0.01, *t*-test). We further assessed the role of endogenous NO in the promoter activity of HO-1. We inhibited endogenous NO synthesis with 1400 W, the more selective inhibitor of iNOS, despite the strong inhibition of HO-1 protein induction by *l*-NMMA. Figure 5A, right panel, shows that pretreatment of cells with 10 µM of 1400 W significantly reduced simvastatin-dependent HO-1 promoter activity by 80±5.2% (n = 3, p<0.02, *t*-test). 1400 W alone had no effect on the promoter activity. 1.3 kbp HO-1 promoter activities were increased dose-dependently by both statins with significant increase at concentrations as low as 1 µM of simvastatin or fluvastatin with an IC_50_ of 1.4±0.3 µM for simvastatin (Figure 5B, left Panel) and 0.6±0.03 µM for fluvastatin (Figure 5B, right Panel). We have previously shown that statins activation of HO-1 involved C/EBPβ and δ as well as USF-1 and USF-2. To determine the transcription factors involved in the response to statins, we performed gel retardation assays in RAW 264.7 cells and eMPM of C57BL/6. Cells were treated with 25 µM simvastatin or 10 µM fluvastatin for 6, 12 or 24 hours and nuclear proteins were subjected to gel retardation assay after incubation with double-stranded (ds) DNA sequences for (HO-1) C/EBP, AP-1 or USF. As shown in figures 6A for RAW 264.7 and 6B for eMPM of C57BL/6, both statins induced protein- DNA complexes for C/EBP binding sequence compared to control at 24 hours. The complex was competed with an excess of cold ds-DNA C/EBP but not with an excess of ds-DNA GATA or USF. Antibodies against C/EBPβ but not against C/EBPα or δ were able to cause a supershift in the C/EBP-protein complex suggesting a role for the βisoform in the promoter activation (Figure 6). Significant binding was also obtained in response to statins at 12- and 24- hours treatment and this binding was competed with an excess of cold AP-1 but not with an excess of the double-stranded oligonucleotides containing the GATA or USF recognition sequence (Figure 6C for RAW 264.7 and Figure 6D for macrophages from C57BL/6). In these cells, gel retardation assays for USF showed a strong basal signal of DNA binding in untreated cells which was not modified upon treatment with statins at different periods of time (n = 3, data not shown).

## Discussion

In this study, we compared the effect of statins on HO-1 expression in 2 murine macrophages cell lines, RAW 264.7 and J774A.1, and in primary macrophages. Because NO is an important cellular mediator in macrophages induced in response to inflammation and since NO induces HO-1 gene, we investigated its role in statin-dependent induction of HO-1. We showed that NO synthase plays a role in some types of macrophages. We first observed an important additive effect between low concentration of LPS and statins on HO-1 expression for all types of macrophages, cell lines and primary macrophages. The results in RAW 264.7 cells are consistent with the data by Leung et al [18]. Since LPS increased NO in a large majority of cells, we investigated blocking endogenous NO synthase activity on HO-1 expression. Our results show that endogenous NO is partially responsible for the statin-dependent upregulation of HO-1 protein in RAW 264.7 and J774A.1 cell lines. We also observed a significant increase in nitrite formation and iNOS expression in response to LPS and the results suggested that NO is a player in the intracellular signal transduction pathways leading to statin-dependent induction of HO-1 in these macrophage cell lines. In RAW 264.7 cells, this result was confirmed by the effect of statins on HO-1 promoter activity. However, in primary peritoneal macrophages, a different role of NO was observed where statin increased HO-1 expression in a NO synthase- independent manner. This was obtained in primary peritoneal macrophages from BALB/c mice that did not produce endogenous NO probably due to a predominant arginase activity in these mice [27], and in macrophages from C57BL/6 mice that have the capacity to produce NO. These observations suggested different mechanisms of regulation of HO-1, which might be due to macrophage subsets and types, the mice models and cell lines [29]. Our results on the role of endogenous NO in statin-dependent HO-1 expression show a different intracellular signaling between macrophages. Although primary macrophages could constitute a better model for murine macrophages than the cell lines RAW 264.7 and J774.1A, a lot of scientists still rely on these cell lines as models of cultured macrophages. Both cell lines were initially prepared from BALB/c mice but reports showed clearly that these cells maintained their capacity to produce NO in response to LPS [30].

In this study, we also investigated the role of p38 MAP kinases in response to statins. p38 MAP kinases were shown to play a central role in HO-1 and in C/EBP induction by many cell regulators [31], [32]. Moreover, statins activate p38 MAP kinase [28]. We showed in the present study that inhibition of p38 MAP kinases decreased HO-1 expression in response to statins in all macrophages whereas intracellular NO is not involved in primary eMPM from BALB/c and C57BL/6 mice. This suggested both common and divergent pathways between the different macrophages for the regulation of HO-1 expression in response to statins with possible interaction.

In the present study, we showed that inhibition of geranygeranylation and not farnesylation, and more particularly RhoA/C is involved in HO-1 expression by statins unlike Chen et al who showed that both inhibition of farnesylation and geranylgeranylation played a role in the induction of HO-1 by statins [20]. We further demonstrated that RhoA/C is a potential target for statins in the regulation of HO-1 expression. Our results are different from the ones described by Grosser *et al.* [33] who showed that statin-dependent activation of HO-1 did not involve inhibition of HMG CoA reductase in ECV 304, a human cell line earlier described as an endothelial immortalized cell line and further characterized as a human bladder cancer cell line [34]. The mevalonate-independent effect was probably due to the very high concentrations of statins (up to 300 µM). However, Ali *et al.* have shown that the increase in HO-1 in human primary endothelial cells in response to statins was mevalonate-dependent [35]. The only significant effect of statin that was mevalonate-independent was by Weitz-Schmidt *et al* who described a unique example where statin inhibits lymphocyte function-associated antigen-1 (LFA-1) interactions with adhesion molecules including ICAM-1 [36], [37]. Our results converge with the mevalonate-dependent type of regulation of HO-1.

We also examined the transcriptional regulation of HO-1 by statins. Previous studies have shown an activation of AP-1 transcription factor in response to statins in RAW 264.7 cells [20] and checked the effect of statins on AP-1 promoter using truncated HO-1 promoters in ECV 304 cell line [33]. We have recently reported a role for C/EBPβ and δ as well as USF-1 and −2 in statin-dependent induction of HO-1 in NIH 3T3 [21]. In the present study, we compared the role of C/EBP isoforms, AP-1 and USF in this more physiologically relevant system and tested for the first time their activation and demonstrated that only C/EBPβ and AP-1 but not C/EBPδ or USF-1/−2 are activated in response to statin. Reporter analysis using 1.3 kbp HO-1 gene proximal promoter accounted for the majority of statin-dependent effect and its activity was strongly reduced in the presence of NO synthase inhibitor and mevalonate. Our results are not supportive of a role of the distal promoter as previously described by Grosser et al in ECV 304 cell line [33]. Thus, regulation of the HO-1 promoter C/EBP in response to statins may differ among the different cell types.

In primary eMPM cells, we showed that simvastatin treatment resulted in reduced IL-6 and TNFα levels. Statin-dependent inhibition of TNFαalthough modest was restored to normal using Sn- protoporphyrin, a selective inhibitor of HO-1 activity, suggesting an involvement of HO-1 in statin-dependent effect on TNFα formation. In macrophages/monocytes, numerous reports suggest a participation of HO-1 in the resolution of inflammation [15]. HO-1 is expressed in response to many proinflammatory cytokines and in many inflammatory settings [9], [10], [38]. Future investigation in *in vivo* models of inflammation will help confirm the protective anti-inflammatory role of HO-1 mediated by statins. Statins have been attributed many anti-inflammatory effects as part of their beneficial pleiotropic effects. This included decrease in reactive oxygen species, NADPH oxidase, matrix metalloproteinases, adhesion molecule expression and cytokine formation [39], [40], [41]. Very few studies addressed the regulation of HO-1 by statins in human macrophages. Recently, Gueler *et al* described a role of statin- induced HO-1 in protection against kidney failure [42]. HO-1 is induced in atherosclerotic plaques [43] and in dendritic cells [44] and played a role in protection against injury.

Although the anti-inflammatory and anti-oxidant effects of statins in our study and others [45] were obtained at higher therapeutic plasma circulating dose, the regulatory effect was specifically targeting inhibition of HMG CoA reductase without toxicity. This was supported by the effect of mevalonate, the direct product of HMG CoA reductase and the isoprenoid-derivatives, which reversed the induction of HO-1 by statins. Studies conducted *in vitro* and *in vivo* helped despite using high concentration of drugs to understand the mechanistic underlying the protective effects of statins in patients with high risk of cardiovascular diseases and vascular inflammation [8].

In summary, we have compared the role of NO in statin-dependent induction of HO-1 in different models of macrophages. The mechanisms behind the different role of NO in statin induction of HO-1 will need future investigation. Our data suggest and implicate a strong transcriptional activity which involved C/EBPβ and AP-1 but not USF-1/−2 or C/EBPδ in response to statins and support anti-inflammatory effects of statins and HO-1 induction.
